# Rapidly progressing renal cell carcinoma associated with Xp11.2 translocations: a case report

**DOI:** 10.1186/1752-1947-6-164

**Published:** 2012-06-27

**Authors:** Akihiro Morii, Yasuyoshi Fujiuchi, Kazuhiro Nomoto, Akira Komiya, Hideki Fuse

**Affiliations:** 1 Department of Urology, Graduate School of Medicine and Pharmaceutical Sciences for Research, University of Toyama, 2630 Sugitani, Toyama, 930-0194, Japan; 2 Diagnostic Pathology, Graduate School of Medicine and Pharmaceutical Sciences for Research, University of Toyama, 2630 Sugitani, Toyama, 930-0194, Japan

## Abstract

**Introduction:**

Renal cell carcinoma associated with Xp11.2 translocations is frequently reported in children, but adult-onset is rare. Here, the case of an adult male who developed a renal cell carcinoma associated with Xp11.2 translocations is presented.

**Case presentation:**

A 38-year-old Asian man presented with left back pain and macroscopic hematuria. Computed tomography revealed a left renal tumor (T3N2M0), and a left radical nephrectomy was performed. Hematoxylin-eosin staining revealed papillary architecture and clear or eosinophilic cytoplasm, and the diagnosis of renal cell carcinoma associated with Xp11.2 translocations/*TFE3* gene fusion was made by the immunohistochemical determination of transcription factor E3 protein. In spite of adjuvant therapy with α-interferon, a recurrent tumor was found in his left lung by computed tomography three months after the nephrectomy. Interleukin-2, tyrosine kinase inhibitors and mammalian target of rapamycin inhibitors showed no effect on tumor progression.

**Conclusions:**

Renal cell carcinomas associated with Xp11.2 translocations have an aggressive clinical course in adults. Strict diagnosis using the immunohistochemistry of transcription factor E3 protein is important to predict the prognosis of such patients and new strategies need to be determined to treat patients with these tumors

## Introduction

Renal cell carcinoma (RCC) associated with Xp11.2 translocations has recently been discovered and integrated into the World Health Organization classification [1]. This type of tumor frequently occurs in children, but adult-onset cases have only lately been reported [2,3]. There are few such reports, and little is known about the clinical course and biological characteristics of this tumor. A case of adult-onset RCC associated with p11.2 translocations is here reported.

## Case presentation

**Figure 1:**
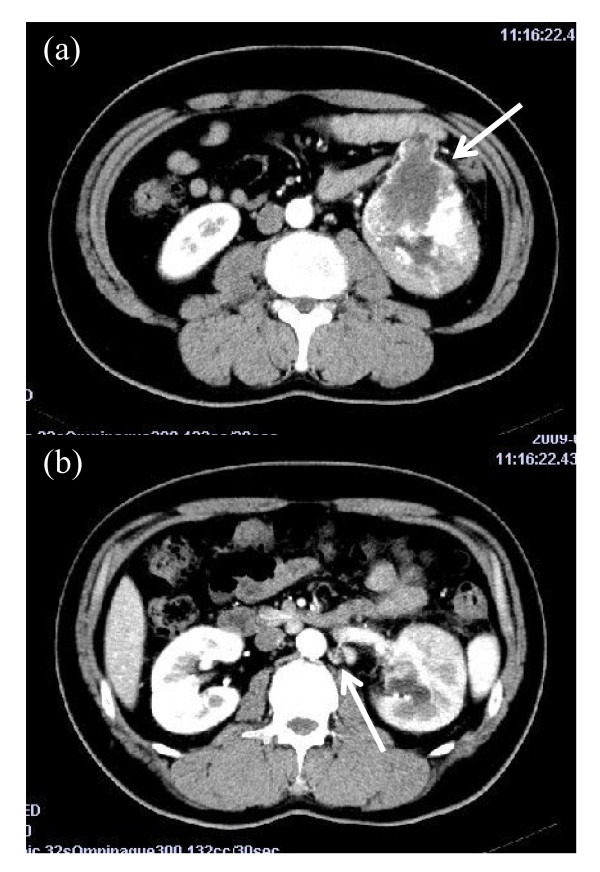
**Abdominal enhanced computed tomography scan at diagnosis. (a)** A tumor of the left kidney measuring 7.2 × 6.5cm (arrow); (**b**) lymph node swelling in the renal hilar region (arrow).

A 38-year-old Asian man presented with macroscopic hematuria and left back pain. He underwent computed tomography of his abdomen and a large enhancing left renal mass and renal hilar lymph node swelling were noted (Figure 1). The tumor was located in the middle-inferior portion of his left kidney. He had no previous history of chemotherapy. All his blood test results were unremarkable. A radical left nephrectomy was performed and the lymph nodes were simultaneously removed.

An ill-demarcated tumor measuring 6 × 6 × 7.5cm was observed in the middle-inferior pole of his left kidney. The cut surface was yellow or white in color. There was hemorrhage and necrosis present.

**Figure 2:**
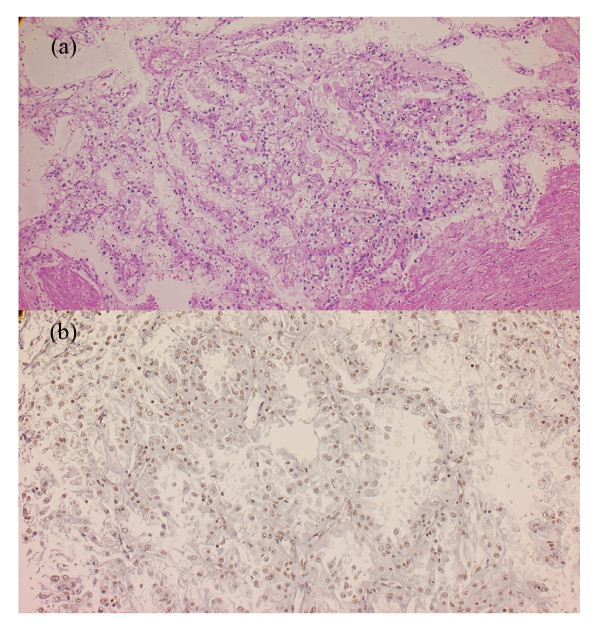
**Histological finding for left renal tumor. (a)** Hematoxilin and eosin staining: the cytoplasm was clear or eosinophilic, and tumor cells proliferated with a papillary architecture or solid pattern; **(b)** transcription factor E3 immunohistochemical labeling of the left renal tumor: nuclei of many tumorous cells were diffusely positive for transcription factor E3.

On microscopy, the tumor consisted of a combined epithelial and sarcomatous component. The epithelial component comprised neoplastic cells with clear or eosinophilic cytoplasm (Figure 2a). Regarding the architectural aspects, the epithelial component had a solid growth pattern or a papillary growth pattern with delicate fibrovascular cores. The neoplastic epithelial cells had enlarged nuclei with an irregular nuclear membrane and distinct nucleoli. This nuclear atypia corresponded to Fuhrman Grade 3. The sarcomatous component consisted of spindle cells with fibroconnective stroma. The neoplastic sarcomatous cells had enlarged irregular nuclei with distinct nucleoli. This nuclear atypia corresponded to Fuhrman Grade 3 to 4. These findings were consistent with sarcomatoid change.

Immunohistochemistry revealed that the nuclei of many tumor cells were positive for transcription factor E3 (TFE3) (Figure 2b). In addition, the neoplastic epithelial cells were diffusely positive for alpha-methylacyl-coenzyme A racemase, CAM5.2 and EMA, and focally positive for cluster of differentiation 10 and vimentin. The neoplastic sarcomatous cells were focally positive for alpha-methylacyl-coenzyme A racemase, EMA and vimentin. The tumor cells, however, were negative for cytokeratin 7, Melan A and human melanoma black-45. Therefore, the tumor was finally diagnosed as RCC associated with Xp11.2 translocation/*TFE3* gene fusion. Renal vein involvement was demonstrated, but lymph node metastasis and distant metastasis were absent. Accordingly, the tumor was classified as pT2pN0M0, Stage II.

Alpha-interferon was administered as adjuvant therapy after the surgery. A recurrent mass was found in his left lung by computed tomography three months after surgery, in spite of the adjuvant therapy. The tumor enlarged despite treatment with interleukin-2. Tyrosine kinase inhibitors sunitinib and sorafenib and the mammalian target of rapamycin inhibitor everolimus were sequentially administered, but the tumor showed no response. Metastatic tumors developed in our patient’s brain, liver and bone and he died 16 months after the nephrectomy.

## Discussion

RCC associated with Xp11.2 translocations accounts for approximately 5% to 20% of RCCs in pediatric and adolescent patients, but it is much less common in adults [4]. Five patterns of fusion with the *TFE3* gene, with *APSLPRCCPSFNonO* and *CLTC* genes, have been found [2,5]. There is little data concerning the mechanism and factors associated with this tumor.

These tumors have an aggressive clinical course in adults [5]. They show poor prognosis, owing to the lack of effective therapy apart from surgery. Moreover, many patients already have local invasion and/or metastasis at the time of diagnosis [4]. Argani *et al*. reported that 14 of 28 patients with adult-onset Xp11 translocation RCC presented at stage IV, whereas metastatic carcinoma involving the lymph nodes occurred in 11 of 13 cases, in whom the lymph nodes were resected [2]. Meyer *et al*. suggested that it was possible that translocation RCCs develop when patients are young, but the tumors are not detected until reaching an advanced stage [6].

Chemotherapy, including the molecularly-targeted drugs α-interferon and interleukin-2 [3,6,7], is used to treat these tumors but have no effect. Choueiri *et al*. reported the outcome of 12 patients with RCC associated with Xp11.2 translocations undergoing anti-vascular endothelial growth factor therapy, three of whom achieved a partial response. They concluded that vascular endothelial growth factor-targeted agents appeared to demonstrate some efficacy for these patients [8]. Alpha-interferon, interleukin-2, sorafenib, sunitinib and everolimus were administered to our patient, but tumor progression could not be suppressed.

The histological features that are useful in differentiating RCC associated with Xp11.2 translocations from the other types of RCC include the combinations of nested and papillary architecture, clear cytoplasm, and extensive psammomatous calcifications. However, these morphological features may overlap with other more common types of RCC. The most distinctive immunohistochemical marker of RCC associated with Xp11.2 translocations is detectable nuclear staining for TFE3 protein. All TFE3 fusion proteins retain the C-terminal portion of TFE3. The RCC in the current patient showed papillary architecture and clear cytoplasm, and the tumor was diagnosed by immunohistochemical findings of TFE3 protein. Strict diagnosis using the immunohistochemistry of TFE3 may increase the number of cases of RCC associated with Xp11.2 translocations that are reported.

Recently, Tsuda *et al*. [9] demonstrated that TFE3 increases Met protein expression in cellular cancer cell lines, including in renal carcinoma. They revealed the responsiveness of an Xp11 translocation RCC cell line to a Met inhibitor. Sagara *et al*. [7] reported the case of a patient with TFE3-renal carcinoma with strong expression of pY1234/1235 hepatocyte growth factor receptor/Met. The hepatocyte growth factor receptor/Met signaling pathway stimulates cell proliferation and migration in many cancers [10]. This could be a therapeutic target and further investigations might discover new strategies to treat patients with these tumors.

## Conclusions

We present a case of an adult-onset RCC associated with Xp11.2 translocations. These tumors have an aggressive clinical course in adults, and there is no effective treatment. Strict diagnosis using the immunohistochemistry of TFE3 is important to predict the prognosis of such patients and new strategies are needed to treat patients with these tumors.

## Consent

Written informed consent was obtained from the patient’s family for publication of this manuscript and accompanying images. A copy of the written consent is available for review by the Editor-in-Chief of this journal.

## Competing interests

The authors declare that they have no competing interests.

## Authors’ contributions

AM analyzed and interpreted the patient data and wrote the manuscript. YF worked up the clinical details and helped to prepare the manuscript. KN made the histopathological and immunohistochemical diagnosis. AK helped to gather the data and write the manuscript. HF was a major contributor in writing the manuscript. All authors read and approved the final manuscript.
